# Prescription practices for antipsychotic medication in an acute inpatient ward: Risks / benefits discussions with patients and performance of baseline investigations as per NICE guidelines

**DOI:** 10.1192/j.eurpsy.2024.297

**Published:** 2024-08-27

**Authors:** M. Elesawy, G. J. Munday, N. Lekka

**Affiliations:** Sheffield Health and Social Care Foundation Trust, Sheffield, United Kingdom

## Abstract

**Introduction:**

Antipsychotic medications are essential for managing psychiatric disorders, offering symptom relief and improved functioning. However, their use carries potential risks. Vigilant monitoring and patient engagement are crucial when initiating antipsychotics.

**Objectives:**

To explore whether comprehensive discussions with patients concerning the benefits and risks of antipsychotics are taking place, and whether baseline investigations are performed before initiation of antipsychotics as per NICE guidelines.

**Methods:**

This retrospective study focused on admissions to a male acute ward between March 1 and May 31, 2023. Data from electronic patient records included demographics, Mental Health Act (MHA) status, psychiatric diagnoses, medical co-morbidities, documentation of medication benefits / side effects discussions with patients, medication information provision, as well as baseline investigations involving weight, height, waist/hip circumference, pulse, blood pressure, blood glucose, HbA1c, lipid profile, prolactin levels, movement disorder assessment, nutritional status, diet, physical activity, and ECG.

**Results:**

Among 23 admitted patients, 15 were newly initiated or reintroduced to antipsychotics, with 14(93.3%) admitted under the MHA. Primary diagnoses included Paranoid Schizophrenia (33.33%), Unspecified Non-Organic Psychosis (20%), Bipolar Affective Disorder (20%), and others. Medical comorbidities were observed in 10(66.7%), notably type 2 diabetes (40%). Among initiates, 6(40%) were new to treatment, while in 9(60%) it was re-initiated.

Within the 15-patient group, discussions on treatment benefits engaged 11(73.3%), while 4(26.7%) lacked complete documentation. Only 6(40%) had discussions about side effects. Metabolic side effects were discussed with 3(20%), extrapyramidal effects with 2(13.33%), while 4(26.7%) had general side effect talks. Patient information leaflets were given to 5(33.3%) patients. Baseline measurements: 12(80%) had weight/height, 4(26.7%) waist/hip, and 14(93.3%) pulse/blood pressure assessed. Blood tests were declined by 4(26.7%). Baseline glucose, HbA1c, lipids, and prolactin were assessed in 8(53.3%), 10(66.7%), 9(60%), and 8(53.3%) patients respectively. Nutritional status was assessed in 13(86.7%), movement disorders in 1(6.7%), and physical activity in 1(6.7%). All 15(100%) patients were offered ECGs, although 5(33.3%) declined.

**Conclusions:**

Adherence to NICE guidelines for baseline investigations and risk/benefit discussions prior to antipsychotic initiation was low, apart from ECGs, weight/height, pulse/blood pressure, assessment of nutritional status, and discussions about antipsychotics’ benefits. Implementing effective monitoring and patient engagement to mitigate potential side effects, is crucial to facilitating the safe and efficient utilisation of antipsychotics.

**Disclosure of Interest:**

None Declared

